# The Relationships Between Biological Activities and Structure of Flavan-3-Ols

**DOI:** 10.3390/ijms12129342

**Published:** 2011-12-13

**Authors:** Cornelia Braicu, Valentina Pilecki, Ovidiu Balacescu, Alexandru Irimie, Ioana Berindan Neagoe

**Affiliations:** 1 Department of Functional Genomics and Experimental Pathology, Cancer Institute “Ion Chiricuta”, 34-35 Republicii, Cluj-Napoca 400015, Romania; E-Mails: cornelia.braicu@iocn.ro (C.B.); ovidiubalacescu@iocn.ro (O.B.); 2 Faculty of Biology and Geology, Babes-Bolyai University, 44 Gheorghe Bilascu, Cluj-Napoca 4000006, Romania; E-Mail: valentinapilecki@yahoo.com; 3 Department of Surgical Oncology, University of Medicine and Pharmacy, “Iuliu Hatieganu”, 8 Victor Babes, Cluj-Napoca 400427, Romania; E-Mail: airimie@umfcluj.ro; 4 Department of Surgery, Cancer Institute “Ion Chiricuta”, 34-35 Republicii, Cluj-Napoca 400015, Romania; 5 Department of Immunology, University of Medicine and Pharmacy “Iuliu Hatieganu”, 8 Victor Babes, Cluj-Napoca 400427, Romania

**Keywords:** catechins, structure-activity relationship, gallate moiety, cell proliferation, apoptosis, pro/antioxidant

## Abstract

Flavan-3-ols are involved in multiple metabolic pathways that induce inhibition of cell proliferation. We studied the structure-activity relationship of gallic acid (GA) and four flavan-3-ols: epigallocatechin gallate (EGCG), epigallocatechin (EGC), catechin (C), and epicatechin (EC). We measured the cell viability by the MTT assay and we determined the concentration of testing compound required to reduce cell viability by 50% (IC_50_). All tested compounds showed a dose-dependent and time-dependent inhibitory antiproliferative effect on Hs578T cells; IC_50_ values varying from the 15.81 to 326.8 μM. Intracellular ROS (reactive oxygen species) were quantified using a fluorescent probe 2′,7′-dichlorofluorescin diacetate (DCFH-DA). Only the treatment with 10 μM EGC and EGCG was able to induce a significant decrease of ROS concentration and increased levels of ROS were registered for 100 μM EGCG, EGC and GA. Flavans-3-ols and GA induced apoptosis in a dose- and time-dependent manner, which indicated that the induction of apoptosis mediated their cytotoxic activity at least partially. The galloylated catechins have shown a stronger antiproliferative activity and apoptotic effect than the one produced by non galloylated catechins. The galloylated flavan-3-ols are potential therapeutic agents for patients with triple negative breast cancer via induction of apoptosis.

## 1. Introduction

**Figure 1:**
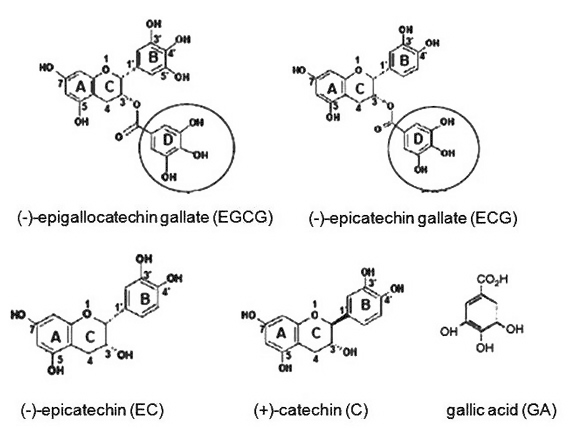
Chemical structure of catechins and gallic acid (GA); the gallate moiety is circled.

The class of natural compounds with biological active properties such as polyphenols has attracted attention in terms of beneficial effects on human health, due to their low toxicity, low cost and high availability. Catechins and catechin gallates belong to the class of polyphenol compounds, subclass of flavan-3-ols, which include (−)-epigallocatechin-3-gallate (EGCG), (−)-epigallocatechin (EGC), (−)-epicatechin (EC), (+)-catechins (C) and (−)-epicatechin-3-gallate (ECG). EGCG and ECG contain a gallic acid moiety at position 3 on the C ring (Figure 1) [1–7].

Flavan-3-ols are characterized by hydroxylated aromatic rings. They are 3-ring phenolic compounds with a double ring attached by a single bond to a third ring and they have multiple hydroxyl groups on the A, B and C rings. The heterocyclic benzopyran ring is known as the C ring, the fused aromatic ring as the A ring, and the phenyl constituent as the B ring. The A ring can be of two types: a phloroglucinol type that is *meta-*trihydroxylated, or a resorcinol type that is *meta*-dihydroxylated. The B ring can be monohydroxylated, *ortho*-dihydroxylated or vicinal-trihydroxylated [1–4]. The catechin and epicatechin are epimers. The structural difference between EGC and EC is an additional hydroxyl group at 5′ position of the B ring for EGC. EGCG is an EGC ester derivative, resulting from an esterification at 3 hydroxyl position of the C ring with a gallate moiety [2,3].

Flavan-3-ols present antioxidant activity in cell-free systems, both *in vitro* and *in vivo*. Many mechanisms of action for the flavan-3-ols chemopreventive activities have been proposed [2,4–12]. One of these mechanisms is their pro/antioxidant activity. The antioxidant capacity of flavan-3-ols is proved particularly by their abilities to upregulate antioxidant enzymes and to scavenge the reactive oxygen species (ROS) [2,6].

Flavan-3-ols and their derivatives are a class of phenolic compounds found in green tea leaves, chocolate, grape and grape seeds, having several healthy properties. An anticarcinogenic activity and a protective capacity against oxidative stress-related diseases were previously described [2,3,10]. Several flavan-3-ols have been reported as apoptosis inducers and inhibitors of cell proliferation in human tumor cells [2,4]. In addition, recent studies indicated their involvement in the modulation of signal transduction pathways, cell survival/death, mitochondrial function and angiogenesis [2–4,6–12].

Most studies have evaluated the biological active properties of EGCG, and only few focused on the relation between the chemical structure and the antiproliferative or anti/prooxidant proprieties of flavan-3-ols, with or without gallate moiety. The development of structure-activity relationships may facilitate the research on cancer therapy. In the present study, we addressed the relation between gallic acid (GA) and flavan-3-ols structures and their activity in Hs579T cell line, an *in vitro* system for highly invasive triple negative human breast cancer [3]. We determined the cell viability and the concentration of each compound required to reduce cell viability by 50% (IC_50_). The putative antiapoptotic and anti/prooxidant properties were investigated in order to evaluate a possible relation between structure and activity of flavan-3-ols and GA.

## 2. Results and Discussion

### 2.1. The Antiproliferative Effect of Flavan-3-Ols and GA

**Figure 2:**
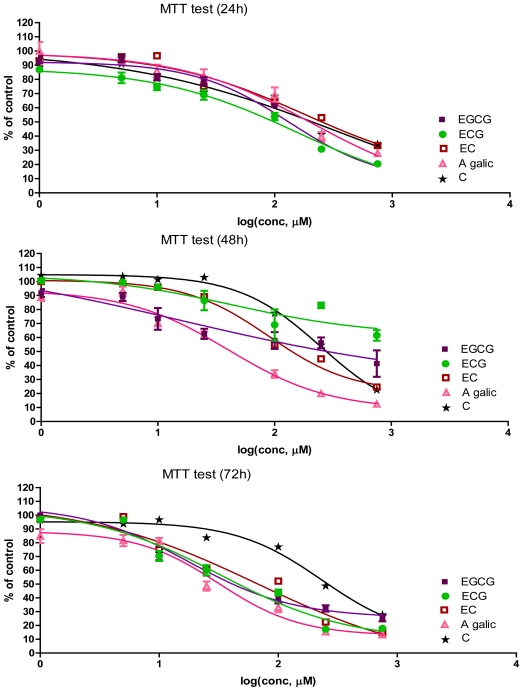
The antiproliferative effect as measured by MTT assay after 24, 48 and 72 h, in incubation with different concentrations (0–750 μM) of flavan-3-ols or GA on Hs578T cell line; log (conc, μM) = log[concentration of bioactive compound, μM] (mean ± SD, n = 6).

At first, we investigated the effect of GA and four types of flavan-3-ols on cultured Hs578T cells. The cells were incubated with the selected compounds, at concentrations between 0–750 μM (Figure 2). The MTT values after 24, 48 and 72 h incubation are represented as % of control, in relation with the log (concentration, μM) (Figure 2). From these plots, IC_50,_ as well as other statistical parameters, were determined using GraphPad Prism free-trial software (Table 1). The results are presented in Figure 2 and Table 1. A difference of the antiproliferative effect based on the measurement of IC_50_ was observed in the case of cellular treatment and was ranked as follows: EGCG > ECG > EC > GA > C as shows 24 hours’ results.

The concentration of GA required to reduce Hs578T cell viability by 50% (IC_50_) was 211.1 μM at 24 h, 40.14 μM at 48 h, and 31.76 μM at 72 h. These results emphasize the fact that gallate moiety is a key component of the antiproliferative effect of tested catechins, by comparing their IC_50_. The highest antiproliferative effect was observed in the case of EGCG, IC_50_ of 131.6 μM at 24 h, 15.8 μM at 48 h, and 17.75 μM at 72 h; C was less efficient.

Our data showed that all compounds tested exert a dose and time-dependent antiproliferative effect on Hs578T cells. However, we found a differential effect for each compound, with IC_50_ values varying from the 15.81 to 326.8 μM. Among the structurally related catechins, the EGCG was the most powerful in inhibiting the growth of breast cancer cells.

Several studies have evaluated the antiproliferative effect of EGCG, but the other flavan-3-ols with/without gallate moiety received less scientific attention. The present study shows that the gallate moiety is a key component. The EGCG contains two gallate moieties, which could explain the highest antiproliferative effect observed in the present study.

After 48 h cell treatment with flavan-3-ols and GA, the MTT values obtained were lower than those observed at 24 h and relatively similar to those registered at 72 h. These results could be explained by their transformation into new metabolic products that induce a higher antiproliferative activity as opposed to the untransformed compound. A similar study [7] demonstrated that some tested phenolic acids exert a dose and time-dependent inhibitory antiproliferative effect on T47D breast cancer cells. Nevertheless, they found a differential effect for each phenolic acid, with IC_50_ values varying between nanomolar and picomolar range [6]. In our study, the flavan-3-ols show various IC_50_ values, within the μM range.

Similar differential growth inhibitions were also observed between the human colorectal cancer cell line (Caco-2) and Hs578T, but not in their respective normal counterparts [8]. The proliferation and/or viability of cultured Hs578T and MDA-MB-231 estrogen receptor-negative breast cancer cell lines was proved to be reduced by EGCG treatment in a similar study. Similar negative effects on proliferation were observed with the DMBA-transformed D3-1 cell line. In 1998, Chen *et al*. evaluated the antiproliferation ability of EGCG in HT-29 cancer cell line. In this study, EGCG inhibited cell proliferation in a dose-dependent manner [6,8]. After 36 h treatment, EGCG inhibited HT-29 cell growth with an IC_50_ of approximately 100 μM [7]. From Figure 2, it can be observed that the EGCG have the best antiproliferative action at all intervals of time evaluated. The IC_50_ values for EGCG at 24 h are in agreement with Chen *et al*., 1998, but at 48 h they are much lower.

The difference between EGCG on one side and the C and EGC on the other side is determined by the gallate group; we can suppose that at 24 h the EGCG treated cells have the best antiproliferative activity due to this gallate. The structural differences in flavan-3-ols might explain the differences in the antiproliferative responses. At 48 h, EGCG and other catechins maintained their highest antiproliferative effects with the highest values than those observed at 24 h. Thus, we can assume that after 24 h, the flavan-3-ols were oxidized and the metabolic products were more efficient. This may prove their pro/antioxidant properties [8].

### 2.2. Apoptotic Effects of Flavan-3-Ols and GA

**Figure 3:**
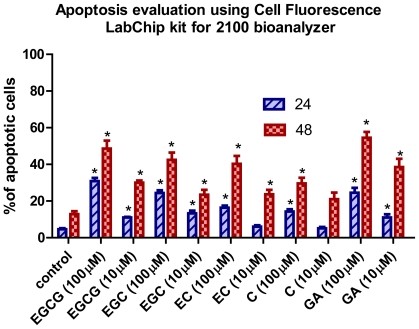
Apoptosis evaluation in Hs578T cells after treatment with flavan-3-ols and GA. The cells were stained with Calcein AM and Annexin-Cy5 and were evaluated using the Cell Fluorescence LabChip kit for 2100 bioanalyzer and the results expressed at % of apoptotic cells at 24 h and 48 h.

The MTT results showed a reduction in cell proliferation, which might be due to an increase in the apoptotic processes. Thus, we measured the apoptosis rate of Hs578T cells exposed to increasing concentrations of different flavan-3-ols (Figure 3).

Cell apoptosis was induced by flavan-3-ols treatment as shown by the flow cytometry (Figure 3). Apoptosis may be induced directly by flavan-3-ols or indirectly by their metabolic products [9]. Our data suggest that the hydroxyl groups on the phenolic ring are essential for the antiproliferative and apoptotic activity. The phenolic hydroxyl groups of catechins are primarily responsible for scavenging free radicals, whereas the galloyl moiety is involved in chelating metal ions [10]. The present study revealed a potentially important role of the galloyl structure in the antiproliferative and proapoptotic responses, when comparing the results obtained for GA with those for the flavan-3-ols.

The intrinsic capacity of catechins to form quinone type metabolites upon their oxidation was demonstrated in a recent study [12]. The formation of quinone type metabolites, especially involving the pyrogallol moiety of these catechins [11], can induce the antiproliferative effect, leading to apoptosis. The pyrogallol-type structure plays an important role in the induction of apoptosis [10,13,14]. These results reveal the fact that flavan-3-ols, such as C and EC lacking the gallate structure, with only catechol and/or resorcinol groups, are less efficient in inducing apoptosis.

The GA induced apoptotic cell death in human promyelocytic leukemia HL-60 cells. The effect of GA was significantly reduced by blocking ROS [15]. This study suggests the prooxidant action of GA in the induction of apoptosis and confirms our data. On the other hand, the addition of larger alkyl groups to the carboxyl group of GA did not increase the ROS either, but significantly increased its apoptosis-inducing activity. Other studies suggested that the capacity to induce apoptosis involves the extracellular production of ROS [16]. This may be due to the increased lipophilicity of the gallate molecule—a factor that induces apoptosis through a yet unknown mechanism [14,15,17].

### 2.3. Detection of Intracellular ROS

**Figure 4:**
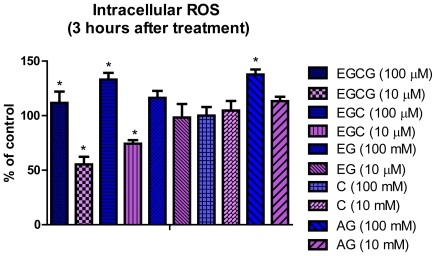
Effect of flavan-3-ols (10 and 100 μM) at 3 h after treatment on evaluation reactive oxygen species (ROS) as measured by DCFH-DA fluorescence intensity on Hs578T cells.

Our unpublished data confirm that the ROS show a steady increase of the fluorescence intensity even in the control group, suggesting that ROS are formed spontaneously in the Hs578T cell line, the maximum fluorescence intensity being registered at 3 h. The effects of two concentrations (10 and 100 μM) of flavan-3-ols and GA at 3 h after treatment are presented in Figure 4. Only the treatment with 10 μM EGC and EGCG was able to induce a significant decrease (p < 0.05) of ROS concentration as compared to the control group and an increased level (p < 0.05) of ROS, as compared to the control group, for 100 μM EGCG, EGC and GA.

Generally, the health promoting activities of catechins, including the antiproliferative effect, are mainly attributed to their antioxidant capacity and ability to scavenge ROS [18]. The antioxidant effects have been proven on several cellular and molecular targets associated with cell death and cell survival [2–4,18]. These properties are due to the presence of the phenolic hydroxy groups on the B ring in ungalloylated catechins (EC and EGC) (Figure 1) and on the B and D rings of the galloylated catechins (ECG and EGCG). The presence of the 3,4,5-trihydroxy B ring has been shown to be significant for the antioxidant and radical scavenging activities, and the order of their effectiveness beeing EGCG > EGC > EC > C.

Flavan-3-ols are highly hydrophilic molecules and there are supposed to act as antioxidants in the aqueous compartment [19]. Antioxidant activity of EGCG at physiological concentrations (0.1–1 μM) [20], underline the interdependency between the scavenging of radicals in the hydrophilic and hydrophobic environments [21]. Thus, it is suggested that the presence of the gallate group at the 3 position plays the most important role in their free radical-scavenging abilities and an additional insertion of a hydroxyl group at the 5′ position in the B ring also contributes to their scavenging activities [20,22].

Specific structural components in catechins are reported to be responsible for the different biologic activities, including ROS, as the present study shows [2]. This is confirmed by the present study for all tested compounds at a concentration of 10 μM. Literature data suggest that the number of hydroxyl groups on the B ring contributes significantly to the ROS scavenging of flavan-3-ols [14]. Recently, the view has changed, and flavonoids are now thought to act as direct antioxidants but rather as inhibitors of prooxidant enzymes or as chelators of transition metals that mask prooxidant actions of reactive nitrogen species (NOS) and ROS [23].

The hydroxyl groups from the three different rings also enhance the inhibition of ROS or induce the prooxidant effect, as it is for EGC and GA. This may be explained by the presence of the ortho-trihydroxyl group in the B ring, which is important for scavenging super-oxide anion, whereas the galloyl moiety is responsible for quenching the hydroxyl radicals [24]. Also, the conjugation between A, B and C rings may provide a resonance effect in the aromatic nucleus that may differentially stabilize the phenoxyl radicals of the different flavan-3-ols structures [14,25,26]. The phenoxyl radicals formed during oxidation are quite stable at higher pH [26].

A large number of studies indicate that tea catechins can act as prooxidants [27,28]. These prooxidant effects are observed under *in vitro* conditions at pharmacological dose [29]. However, it is still unclear whether the effects exerted on molecular endpoints in signal transduction pathways are downstream events of the modulation of pro/antioxidant balance in cells or they are due to the direct action of EGCG and other catechins on the various molecular targets, independently of the antioxidant activities. Furthermore, most of the putative molecular mechanisms that have been proposed are based on *in vitro* studies at far higher EGCG concentrations than those achievable *in vivo*. It has been established that the B ring, hydroxyl group and galloyl moiety of the catechins are the main contributing factors to their scavenging activities and the presence of the ortho-dihydroxyl group in the B ring and the galloyl moiety are important in maintaining the effectiveness of the radical scavenging ability [29]. Our previous studies have shown that physiologically achievable concentrations of flavan-3-ols protect the cells from ROS induction by mycotoxins [14, 29].

## 3. Experimental Section

### 3.1. Cell Culture

HS578T cells (ECACC) have been grown at 37 °C in a humidified atmosphere with 5% CO_2_ in Dulbecco’s Modified Eagle’s Medium (DMEM, Sigma-Aldrich, Germany), with 4500 mg/L glucose, supplemented with 10% fetal bovine serum, without sodium pyruvate, 2 mM L-glutamine, 100 U/mL penicillin and 100 U/mL streptomycin, 10 μg/mL insuline (Actrapid, Novo Nordisk, Denmark).

### 3.2. Cell Treatment

Cells were treated for 24, 48 and 72 h, with different concentrations (1, 5 10, 25, 100, 250, 750 μM) of flavan-3-ols (EGCG, ECG, C, EC) or GA, for the evaluation of cell viability by using the MTT test. All reagents were purchased from Sigma-Aldrich. The evaluation of apoptosis was done at 24 and 48 h after treatment, with 10 and 100 μM of the selected compounds.

### 3.3. MTT Assay

2 × 10^4^ Hs578T cells were treated with flavan-3-ols in a 98-well plate, as described above and incubated for 24, 48 and 72 h, respectively. In order to perform the MTT assay, the culture medium was removed; cells were washed with P BS and 150 μL Hanks salt containing MTT (Sigma-Aldrich, Germany). A final concentration of 455 μg MTT/mL Hanks salt was added into each well. After 2 hour-incubation under standard conditions, the MTT solution was removed a nd 2 00 μL of DMSO were added into each well. The absorbance was measured at 490 nm using a Biotek Synergy HT Microplate Plate Reader.

### 3.4. Detection of Intracellular ROS

Intracellular ROS were quantified by using a fluorescent probe 2′7′-dichlorofluorescin diacetate (DCFH-DA). The DCFH-DA diffuses quickly through the cell membrane and it is enzymatically hydrolyzed by intracellular esterases to non-fluorescent dichlorofluorescin-diacetate (DCFHDA), which is rapidly oxidized to highly fluorescent dichlorofluorescin (DCF) in the presence of intracellular reactive oxygen species. Cells were seeded in 96-well black plates, 2 × 10^3^ cells per well, and allowed to grow for 24 h. After that, the cells were treated with various concentrations of flavan-3-ols and of DCFH-DA (final concentration 0.1 μM). The DCF fluorescence intensity was detected 3 h after treatment using a Biotek Synergy HT Microplate Plate Reader with 485 nm emission and 530 nm excitation wavelengths.

### 3.5. On-Chip Flow Cytometry

The cells were treated as described above and cultured for 24 and 48 h. After incubation, the cells were trypsinized, collected, stained with Anexinn V-biotin Apoptosis Detection kit (Calbiochem) and Calcein AM (Invitrogen) and quantified by on-chip flow-cytometry. The number of apoptotic cells was assessed with Agilent Lab-on-a-chip Bioanalyzer 2100, as percent of apoptotic cells in live cells.

## 4. Conclusions

The antiproliferative and proapoptotic effects of the flavan-3-ols and GA are dose and time-dependent. The galloylated catechins showed stronger effects than those of non-galloylated structures. The galloyl moiety appears to be required both for the antiproliferative, apoptotic and antioxidant effects, but there is no clear structure-activity relationship. In conclusion, the results of this study indicate that, from all the dietary flavan-3-ols studied, EGCG is the most efficient in the Hs578T breast cancer cell line. EGCG, EGC or GA are able to induce an inhibition of cell proliferation and to modulate apoptosis. Flavan-3-ols may have a dual function, both as antioxidants and pro-oxidants, depending on their concentration and exposure time on the cell culture.

Further studies in this area are needed to determine if catechins or their metabolites are primarily responsible for the health benefits of flavan-3-ols. Moreover, future studies should be based on the investigation of the precise molecular action mechanisms of flavan-3-ols and GA in Hs578T cells. These studies will bring new data on future therapeutic applications and chemopreventive effects in triple negative breast cancer.

## Figures and Tables

**Table 1:** IC_50_ values determined by the MTT test after 24, 48 and 72 h of treatment on Hs578T cell line.

Time (h)	Compound	IC50 (μM)	Slope	R2
24	EGCG	131.6	−1.086	0.9522
	ECG	155.1	−0.7287	0.9601
	EC	180.4	−0.7303	0.7820
	C	326.8	−0.4809	0.6794
	GA	211.1	−0.7720	0.9016
48	EGCG	15.8	−0.3760	0.6944
	EGC	43.49	−0.7227	0.5283
	EC	89.59	−1.220	0.9251
	C	250.0	−1.233	0.9324
	GA	40.14	−1.002	0.9424
72	EGCG	17.75	−0.9903	0.9509
	ECG	32.56	−0.7985	0.9446
	EC	67.45	−0.5975	0.8716
	C	233	−1.205	0.9136
	GA	31.67	−1.270	0.9394
